# Skyrmion crystals in centrosymmetric itinerant magnets without horizontal mirror plane

**DOI:** 10.1038/s41598-021-90308-1

**Published:** 2021-05-27

**Authors:** Ryota Yambe, Satoru Hayami

**Affiliations:** 1 Department of Physics, Hokkaido University, Sapporo, 060-0810 Japan; 2 Department of Applied Physics, The University of Tokyo, Tokyo, 113-8656 Japan

**Keywords:** Magnetic properties and materials, Topological matter

## Abstract

We theoretically investigate a new stabilization mechanism of a skyrmion crystal (SkX) in centrosymmetric itinerant magnets with magnetic anisotropy. By considering a trigonal crystal system without the horizontal mirror plane, we derive an effective spin model with an anisotropic Ruderman–Kittel–Kasuya–Yosida (RKKY) interaction for a multi-band periodic Anderson model. We find that the anisotropic RKKY interaction gives rise to two distinct SkXs with different skyrmion numbers of one and two depending on a magnetic field. We also clarify that a phase arising from the multiple-*Q* spin density waves becomes a control parameter for a field-induced topological phase transition between the SkXs. The mechanism will be useful not only for understanding the SkXs, such as that in Gd$_2$PdSi$_3$, but also for exploring further skyrmion-hosting materials in trigonal itinerant magnets.

## Introduction

A magnetic skyrmion, which is characterized by a topologically nontrivial spin texture^1–3^, has been extensively studied in condensed matter physics since the discovery of the skyrmion crystal (SkX) in chiral magnets^4–6^. The SkX exhibits a nonzero topological winding number called the skyrmion number $N_{{\mathrm{sk}}}$, which is defined as $N_{{\mathrm{sk}}}=\sum _{R}\Omega _{R}/4\pi$, where $\Omega _R$ is a skyrmion density related to the solid angle consisting of three spins ${\varvec{S}}_i$, ${\varvec{S}}_j$, and ${\varvec{S}}_k$ on the triangle *R*: $\tan \left( \Omega _{R}/2\right) ={\varvec{S}}_i\cdot ({\varvec{S}}_j\times {\varvec{S}}_k)/(1+{\varvec{S}}_i\cdot {\varvec{S}}_j+{\varvec{S}}_j\cdot {\varvec{S}}_k+{\varvec{S}}_k\cdot {\varvec{S}}_i )$^7^. The study of the SkX has attracted much attention, as the swirling topological magnetic texture owing to nonzero $N_{{\mathrm{sk}}}$ gives rise to an emergent electromagnetic field through the spin Berry phase and results in intriguing transport phenomena and dynamics^8–12^, such as the topological Hall effect^13,14^ and the skyrmion Hall effect^15,16^.

**Figure 1:**
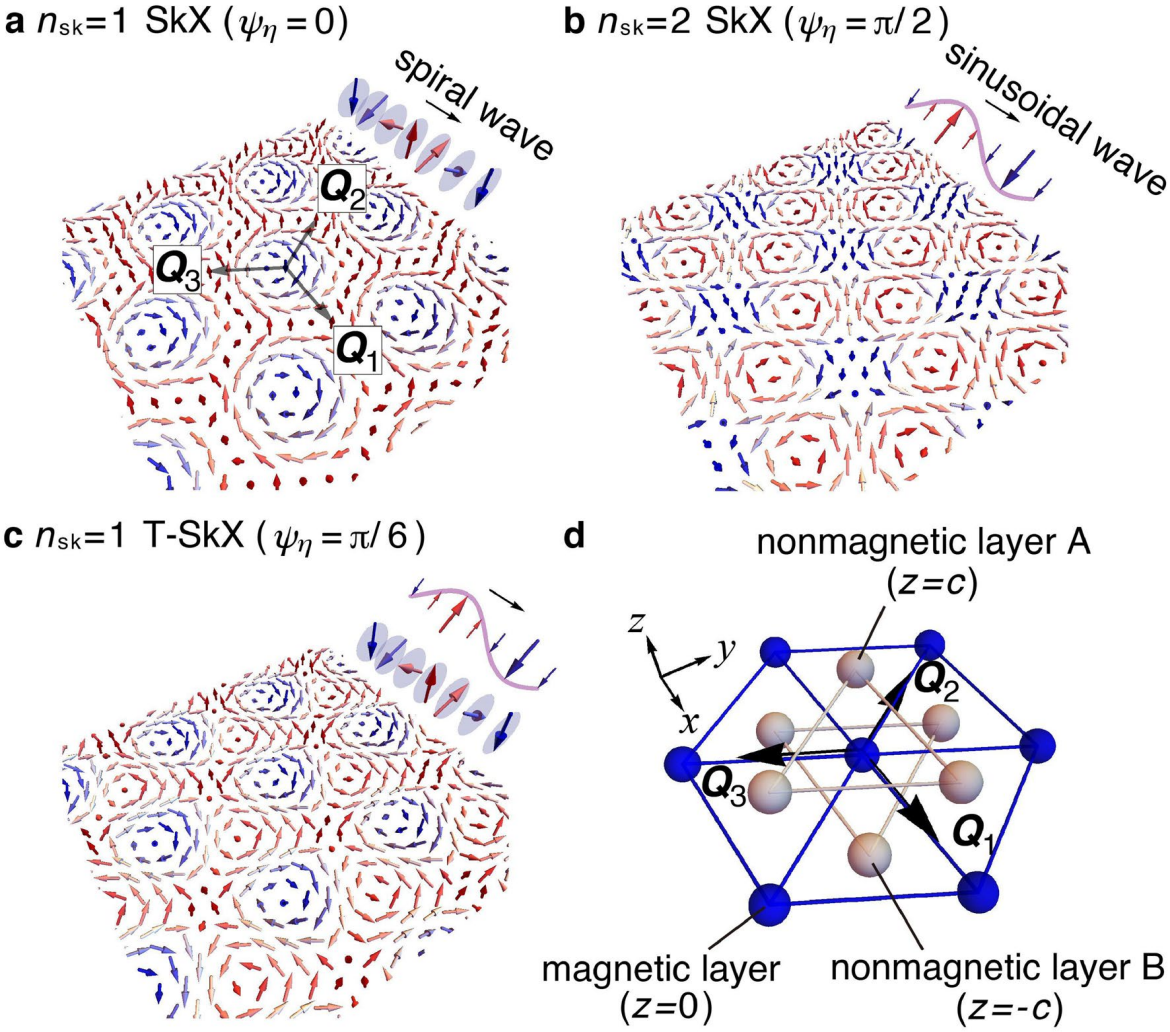
Schematic pictures of the SkXs and the crystal structure. (**a**–**c**) SkXs characterized by three spiral and sinusoidal waves along the ${\varvec{Q}}_1$, ${\varvec{Q}}_2$, and ${\varvec{Q}}_3$ directions: (**a**) the $n_{\mathrm{sk}}=1$ SkX for $\psi _\eta =0$ , (**b**) the $n_{\mathrm{sk}}=2$ SkX for $\psi _\eta =\pi /2$, and (**c**) the $n_{\mathrm{sk}}=1$ T-SkX for $\psi _\eta =\pi /6$ in Eq. (1). (**d**) Centrosymmetric trigonal structure without the horizontal mirror plane. The blue spheres represent magnetic sites, while the gray spheres shifted by $+ c$ ($-c$) from the center of the downward (upward) triangles on the magnetic layer represent nonmagnetic sites on a layer A (B).

The SkXs are expressed as a superposition of three spin density waves (triple-*Q* state) as

1 $$\begin{aligned} {\varvec{S}}_i = \sum _{\eta =1}^3 \left( {\varvec{e}}_{\eta } \sin \mathcal {Q}_{\eta i}'+{\varvec{e}}_{z}\cos \mathcal {Q}_{\eta i}\right) , \end{aligned}$$

where ${\varvec{e}}_{\eta }$ and ${\varvec{e}}_z$ are the unit vectors along the in-plane and *z* directions, respectively. $\mathcal {Q}_{\eta i}={\varvec{Q}}_{\eta }\cdot {\varvec{r}}_i+\phi _{\eta }$, and $\mathcal {Q}_{\eta i}'=\mathcal {Q}_{\eta i}+\psi _\eta$ where $\phi _\eta$ and $\psi _\eta$ are phases of each spin density wave. A variety of the SkXs are described by Eq. (1); a superposition of spiral waves for ${\varvec{e}}_\eta \parallel {\varvec{e}}_z\times {\varvec{Q}}_\eta$ (${\varvec{e}}_\eta \parallel {\varvec{Q}}_\eta$) and ${\psi _1=\psi _2=\psi _3}=0$ or $\pi$ represents the Bloch-type (Néel-type) SkX, while that for $\psi _1=\psi _2=0$ and $\psi _3=\pi$ represents the anti-type SkX. The real-space spin texture for the Bloch-type SkX is shown in Fig. 1a. All the SkXs have the skyrmion number of one, $n_{{\mathrm{sk}}} {\equiv |N_{{\mathrm{sk}}}|}=1$, in the magnetic unit cell and breaks the spatial inversion symmetry irrespective of ${\varvec{e}}_\eta$ and $\psi _\eta$^8^. We call them the $n_{\mathrm{sk}}=1$ SkXs. The $n_{\mathrm{sk}}=1$ SkXs are stabilized by the Dzyaloshinskii-Moriya (DM) interaction^17,18^ in chiral/polar magnets^4,19^ or the competing exchange interactions in frustrated magnets^20–22^.

Meanwhile, the spiral density waves are not necessarily for the formation of the SkX. By considering the superposition of the sinusoidal waves characterized by a different $\psi _\eta$, another type of the SkX can emerge, as shown in Fig. 1b^23,24^. In contrast to the $n_{\mathrm{sk}}=1$ SkX, this spin texture exhibits the skyrmion number of two in a magnetic unit cell ($n_{\mathrm{sk}}=2$ SkX), whose spatial inversion and/or sixfold rotational symmetries are broken depending on $\phi _\eta$ on a discrete lattice. For example, the $n_{\mathrm{sk}}=2$ SkX with $\phi _\eta =\pi$ shown in Fig. 1b has the inversion symmetry, but the $n_{\mathrm{sk}}=2$ SkX with $\phi _1=4\pi /3$, $\phi _2=2\pi /3$, and $\phi _3=\pi$ shows the inversion symmetry breaking. Although the $n_{\mathrm{sk}}=2$ SkX seems to be rare compared to the $n_{\mathrm{sk}}=1$ one, it is stabilized by a multi-spin interaction in itinerant magnets ^23,24^ or an anisotropic symmetric exchange interaction in frustrated magnets^25^. Moreover, an isolated skyrmion with $n_{\mathrm{sk}}=2$ is nucleated in frustrated magnets^26,27^.

In the present study, we report our theoretical discovery of the SkXs by focusing on a magnetic anisotropy that arises from the absence of the mirror symmetry in the crystal structure. By constructing a microscopic effective spin model and performing simulated annealing for triangular itinerant magnets, we show that an anisotropic Ruderman–Kittel–Kasuya–Yosida (RKKY) interaction ^28–30^ arising from the absence of the mirror symmetry on a magnetic layer [Fig. 1d] induces the SkXs with $n_{\mathrm{sk}}=1$ and $n_{\mathrm{sk}}=2$. The anisotropic RKKY interaction stabilizes the SkXs even without the DM, competing exchange, and multi-spin interactions^25,31–35^. The obtained SkXs exhibit different symmetry breaking compared to that found in previous studies^4,19^. The spin texture in the SkX with $n_{\mathrm{sk}}=1$ does not have the sixfold rotational symmetry in addition to the inversion symmetry, as shown in Fig. 1c, which is different from that in chiral and frustrated magnets in Fig. 1a. We here call this state the $n_{\mathrm{sk}}=1$ threefold-rotational-symmetric SkX (T-SkX). Meanwhile, the $n_{\mathrm{sk}}=2$ SkX shows the inversion symmetry breaking. Furthermore, we elucidate that topological phase transitions between the $n_{\mathrm{sk}}=1$ T-SkX, the $n_{\mathrm{sk}}=2$ SkX, and another non-topological triple-*Q* state are caused by a change with respect to the relative phase $\psi _\eta$ in Eq. (1), which is controlled by the degree of the mirror symmetry breaking. This mechanism for the SkXs might be useful to understand a microscopic origin of the SkX in Gd$_2$PdSi$_3$^36–38^, as the underlying lattice structure without the mirror plane on a magnetic layer is common^39^.

## Results

### Model

Let us start by showing an effective spin model starting from a multi-band periodic Anderson model consisting of localized and itinerant electrons. To trace out the itinerant electron degree of freedom and obtain effective magnetic interactions between localized spins, we adopt the standard Schrieffer-Wolff transformation^40^ and perform the perturbative expansion of the grand potential with respect to the anisotropic spin-charge coupling^24,41^, as detailed in Supplementary Information. Generally, the effective spin model is given by

2 $$\begin{aligned} \mathcal {H}&= -2\sum _{\eta }\left[ J_{{\varvec{Q}}_\eta }{\varvec{S}}_{{\varvec{Q}}_\eta }\cdot {\varvec{S}}_{-{\varvec{Q}}_\eta } +\sum _{\alpha \beta } K^{\alpha \beta }_{{\varvec{Q}}_\eta } S^{\alpha }_{{\varvec{Q}}_\eta }S^\beta _{-{\varvec{Q}}_\eta } +i{\varvec{D}}_{{\varvec{Q}}_\eta }\cdot \left( {\varvec{S}}_{{\varvec{Q}}_\eta }\times {\varvec{S}}_{-{\varvec{Q}}_\eta } \right) \right] , \end{aligned}$$

where $\alpha , \beta =x,y,z$, ${\varvec{S}}_{{{\varvec{Q}}}_{\eta }}$ is the Fourier transform of the localized electron spin ${\varvec{S}}_i$ at site *i* ($|{\varvec{S}}_i|=1$), and the coefficient 2 arises from the $-{\varvec{Q}}_\eta$ contribution. The effective spin model consists of an isotropic RKKY interaction, symmetric anisotropic RKKY interaction, and antisymmetric DM-type RKKY interaction with coupling constants $J_{{\varvec{Q}}_\eta }$, $K^{\alpha \beta }_{{\varvec{Q}}_\eta }$, and $D^{\alpha }_{{\varvec{Q}}_\eta }$, respectively. The coupling constants are defined by

3 $$\begin{aligned} J_{{\varvec{Q}}_\eta }&=\frac{1}{3}\sum _{\alpha } \chi ^{\alpha \alpha }_{{\varvec{Q}}_\eta }, \end{aligned}$$

4 $$\begin{aligned} K^{\alpha \beta }_{{\varvec{Q}}_\eta }&=\mathrm{Re}\left[ \chi ^{\alpha \beta }_{{\varvec{Q}}_\eta }\right] -J_{{\varvec{Q}}_\eta }\delta _{\alpha \beta }, \end{aligned}$$

5 $$\begin{aligned} D^{\alpha }_{{\varvec{Q}}_\eta }&=\sum _{\beta \gamma }\frac{\epsilon _{\alpha \beta \gamma }}{2} \mathrm{Im}\left[ \chi ^{\beta \gamma }_{{\varvec{Q}}_\eta }\right] , \end{aligned}$$

where $\chi _{{\varvec{q}}}^{\alpha \beta }$ corresponds to the bare susceptibility of itinerant electrons, $\delta _{\alpha \beta }$ is the Kronecker delta, and $\epsilon _{\alpha \beta \gamma }$ is the Levi-Civita symbol. In Eq. (2), the wave vector ${\varvec{Q}}_\eta$ is chosen by supposing that $\chi _{{\varvec{Q}}_\eta } > \chi _{{\varvec{q}}}$, which is relevant to the lattice symmetry. The anisotropic interactions, $K^{\alpha \beta }_{{\varvec{Q}}_\eta }$, and $D^{\alpha }_{{\varvec{Q}}_\eta }$, originate from the atomic spin-orbit coupling^42–44^. The number of ${\varvec{Q}}_\eta$ and nonzero components of the interactions are determined by the lattice symmetry.

For the above effective spin model, we consider the lattice structure in Fig. 1d consisting of a magnetic layer sandwiched by two nonmagnetic layers. The nonmagnetic ions at $z=c$ ($z=-c$) are located above (below) the downward (upward) triangles on the magnetic layer at $z=0$, which breaks the horizontal mirror symmetry at $z=0$ while keeping the inversion symmetry. The lattice symmetry is compatible with the $D_{\mathrm{3d}}$ point group symmetry. In this situation, we set three ${\varvec{Q}}_\eta$ and four independent coupling constants to satisfy the $D_{\mathrm{3d}}$ symmetry. The former is given by ${\varvec{Q}}_1=(2\pi /6,0,0)$, ${\varvec{Q}}_2=(-\pi /6,\sqrt{3}\pi /6,0)$, and ${\varvec{Q}}_3=(-\pi /6,-\sqrt{3}\pi /6,0)$ and the latter is given by $J_{{\varvec{Q}}_1}$, $K^{xx}_{{\varvec{Q}}_1}$, $K^{yy}_{{\varvec{Q}}_1}$, and $K^{yz}_{{\varvec{Q}}_1}$: $J_{{\varvec{Q}}_1}=J_{{\varvec{Q}}_2}=J_{{\varvec{Q}}_3}$, $K^{xx}_{{\varvec{Q}}_2}=K^{xx}_{{\varvec{Q}}_3}=(K^{xx}_{{\varvec{Q}}_1}+3K^{yy}_{{\varvec{Q}}_1})/4$, $K^{yy}_{{\varvec{Q}}_2}=K^{yy}_{{\varvec{Q}}_3}=(3K^{xx}_{{\varvec{Q}}_1}+K^{yy}_{{\varvec{Q}}_1})/4$, $K^{zz}_{{\varvec{Q}}_1}=K^{zz}_{{\varvec{Q}}_2}=K^{zz}_{{\varvec{Q}}_3}=-K^{xx}_{{\varvec{Q}}_1}-K^{yy}_{{\varvec{Q}}_1}$, $-K^{xy}_{{\varvec{Q}}_2}=-K^{yx}_{{\varvec{Q}}_2}=K^{xy}_{{\varvec{Q}}_3}=K^{yx}_{{\varvec{Q}}_3}=\sqrt{3}(K^{xx}_{{\varvec{Q}}_1}-K^{yy}_{{\varvec{Q}}_1})/4$, $K^{yz}_{{\varvec{Q}}_1}=K^{zy}_{{\varvec{Q}}_1}=-2K^{xz}_{{\varvec{Q}}_2}/\sqrt{3}=-2K^{zx}_{{\varvec{Q}}_2}/\sqrt{3}=-2K^{yz}_{{\varvec{Q}}_2}=-2K^{zy}_{{\varvec{Q}}_2}=2K^{xz}_{{\varvec{Q}}_3}/\sqrt{3}=2K^{zx}_{{\varvec{Q}}_3}/\sqrt{3}=-2K^{yz}_{{\varvec{Q}}_3}=-2K^{zy}_{{\varvec{Q}}_3}$ (all other coupling constants are zero). Among three anisotropic coupling constants, we focus on the effect of $K^{yz}_{{\varvec{Q}}_1}$, which originates from the horizontal mirror symmetry breaking and is characteristic of the $D_{\mathrm{3d}}$ symmetry, on the stabilization of the multiple-*Q* states, and $K^{xx}_{{\varvec{Q}}_1}$ and $K^{yy}_{{\varvec{Q}}_1}$ are neglected for simplicity^31,34^. In the end, the effective spin model is summarized as

6 $$\begin{aligned} \mathcal {H}&=-2\sum _{\eta {=1}}^{{3}} \left( J{\varvec{S}}_{{\varvec{Q}}_{\eta }} \cdot {\varvec{S}}_{-{\varvec{Q}}_{\eta }} +\Gamma \sum _{\alpha \beta }I^{\alpha \beta }_{{{\varvec{Q}}_\eta }}S^{\alpha }_{{\varvec{Q}}_{\eta }}S^{\beta }_{-{\varvec{Q}}_{\eta }}\right) -H \sum _i S_i^z{.} \end{aligned}$$

Here, $J\equiv J_{{\varvec{Q}}_1}$, $\Gamma \equiv K^{yz}_{{\varvec{Q}}_1}$, $I^{yz}_{{\varvec{Q}}_1}$=$I^{zy}_{{\varvec{Q}}_1}$=1, $I^{yz}_{{\varvec{Q}}_2}$=$I^{zy}_{{\varvec{Q}}_2}$=$-1/2$, $I^{xz}_{{\varvec{Q}}_2}$=$I^{zx}_{{\varvec{Q}}_2}$=$-\sqrt{3}/2$, $I^{yz}_{{\varvec{Q}}_3}$=$I^{zy}_{{\varvec{Q}}_3}$=$-1/2$, and $I^{xz}_{{\varvec{Q}}_3}$=$I^{zx}_{{\varvec{Q}}_3}$=$\sqrt{3}/2$ (all other component of $I_{{{\varvec{Q}}_\eta }}$ are zero). The symmetric aniostropic interaction with $\Gamma$ is qualitatively different from the antisymmetric DM interaction: the former can appear irrespective of the inversion symmetry, while the latter requires the inversion symmetry breaking, and thus vanishes in Eq. (6). The $\Gamma$ term also appears in the other trigonal crystal systems. We also introduce the Zeeman coupling to an external magnetic field *H* along the *z* direction.

**Figure 2:**
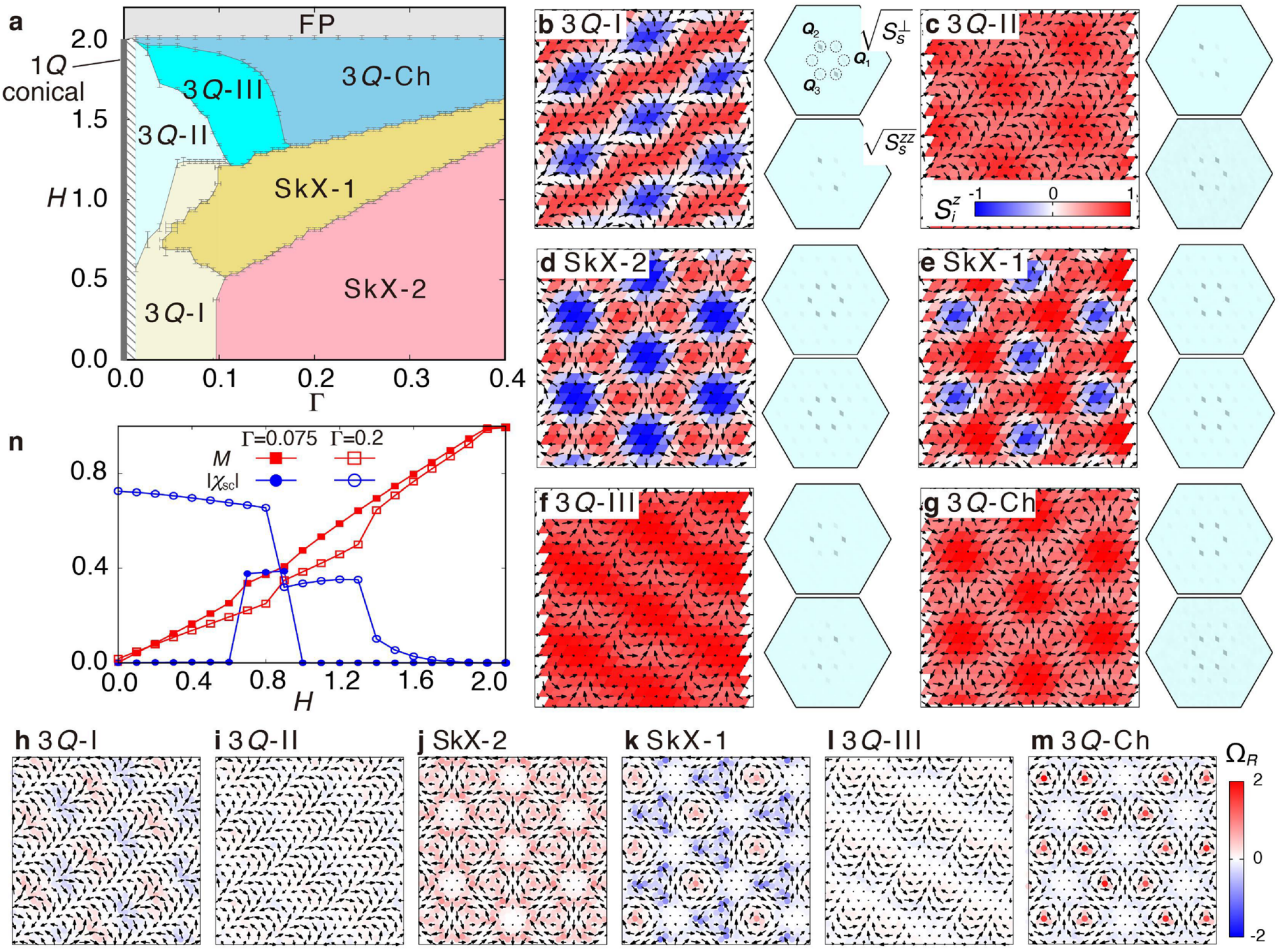
Magnetic phase diagram and characteristics of magnetic phases. (**a**) $\Gamma$-*H* magnetic phase diagram for the model in Eq. (6) in the unit of *J*. The 3*Q*-I, 3*Q*-II, 3*Q*-III, SkX-2, SkX-1, 3*Q*-Ch, 1*Q* conical, and FP represent the triple-*Q* I, triple-*Q* II, triple-*Q* III, $n_{\mathrm{sk}}=2$ SkX, $n_{\mathrm{sk}}=1$ T-SkX, triple-*Q* chiral, single-*Q* conical, and fully polarized states, respectively. In the hatched region, energies for several magnetic states are degenerate and it is difficult to determine the phase boundaries. (**b**–**g**) Snapshots of the spin configurations in (**b**) 3*Q*-I for $\Gamma =0.075$ and $H=0.4$, (**c**) 3*Q*-II for $\Gamma =0.075$ and $H=1.3$, (**d**) SkX-2 for $\Gamma =0.2$ and $H=0$, (**e**) SkX-1 for $\Gamma =0.2$ and $H=1$, (**f**) 3*Q*-III for $\Gamma =0.075$ and $H=1.6$, and (**g**) 3*Q*-Ch for $\Gamma =0.2$ and $H=1.4$. The arrows and contour denote the *xy* and *z* components of the spin moments, respectively. The square root of in-plane and out-of-plane spin structure factors in the Brillouin zone are shown in upper and lower panels, respectively, where the dashed circles highlight $\pm {\varvec{Q}}_1$, $\pm {\varvec{Q}}_2$, and $\pm {\varvec{Q}}_3$ and the ${\varvec{q}}=0$ component is removed for better visibility. (**h**–**m**) Real-space distributions of the skyrmion density $\Omega _R$ for the spin configurations in (**b**–**g**), respectively. (**n**) *H* dependences of the magnetization (red square) and spin scalar chirality (blue circle) for $\Gamma =0.075$ (filled symbols) and $\Gamma =0.2$ (open symbols).

### Magnetic phase diagram

A magnetic phase diagram of the model in Eq. (6) is calculated by simulated annealing combined with the standard Metropolis local updates. Figure 2a shows the magnetic phase diagram while changing $\Gamma$ and *H* in the unit of *J* at a temperature of 0.01. To identify magnetic phases, we compute the magnetization $M=(1/N)\sum _{j}\langle S_j^{z} \rangle$ and the spin structure factor $S_s^{\alpha \alpha }({\varvec{q}})=(1/N)\sum _{jl}\langle S_j^{\alpha }S_l^{\alpha } \rangle e^{i{\varvec{q}}\cdot ({\varvec{r}}_j-{\varvec{r}}_l)}$, where ${\varvec{r}}_j$ is the position vector at site *j*, $N=48^2$ is the system size, and $\langle \cdots \rangle$ is the thermal average. We also calculate the spin scalar chirality $\chi _{\mathrm{sc}}=(1/N)\sum _{R}\langle [ {\varvec{S}}_i\cdot ({\varvec{S}}_j\times {\varvec{S}}_k) ] _R \rangle$ where the subscript *R* represents the center of the triangle and *i*, *j* and *k* are in the counterclockwise order. We obtain six different magnetic phases besides the single-*Q* (1*Q*) conical state for $\Gamma =0$ and the fully-polarized (FP) state for $H \gtrsim 2$, whose real-space spin configurations and the in-(out-of-)plane spin structure factor $S_s^{\perp }({\varvec{q}})=S_s^{xx}({\varvec{q}})+S_s^{yy}({\varvec{q}})$ [$S_s^{zz}({\varvec{q}})$] are shown in Fig. 2b–g. We also show the skyrmion density $\Omega _R$ for each spin configuration in Fig. 2h–m.

For $\Gamma =0$, the model in Eq. (6) reduces to the isotropic RKKY model, which stabilizes the 1*Q* conical state for any *H*. By introducing $\Gamma$, the multiple-*Q* instabilities occur: the triple-*Q* I (3*Q*-I) state is stabilized for small *H*, while the triple-*Q* II (3*Q*-II) state is stabilized for large *H,* as shown in Fig. 2a. Their spin modulations are mainly characterized by the in-plane single-*q* component, which smoothly connects to the 1*Q* conical state. Meanwhile, they exhibit different peak structures in $S_s^{zz}({\varvec{q}})$, as shown in Fig. 2b,c: there is a dominant peak at ${\varvec{Q}}_2$ in the 3*Q*-I state, whereas there are two dominant peaks at ${\varvec{Q}}_1$ and ${\varvec{Q}}_3$ in the 3*Q*-II state in addition to the peak at ${\varvec{Q}}_2$ in $S_s^{\perp }({\varvec{q}})$. Both phases are topologically trivial without $\chi _{\mathrm{sc}}$.

While increasing $\Gamma$, the 3*Q*-I state is replaced by the $n_{\mathrm{sk}}=2$ SkX (SkX-2) in the low-field region for $\Gamma \gtrsim 0.1$ and the $n_{\mathrm{sk}}=1$ T-SkX (SkX-1) in the intermediate-field region for $\Gamma \gtrsim 0.05$, as shown in Fig. 2a. Both SkXs are characterized by the triple-*Q* peaks with the same intensities, as shown in Fig. 2d,e. By looking into the real-space spin configurations, they are formed by a vortex with vorticity $\nu =-2$ and two vortices with $\nu =+1$ in a magnetic unit cell in Fig. 2d,e, which indicates the inversion symmetry breaking. The positions at the cores with negative $S_i^z$ are different with each other. They are located at the cores with $\nu =-2$ ($\nu =+1$) for the $n_{\mathrm{sk}}=2$ SkX ($n_{\mathrm{sk}}=1$ T-SkX). Such a difference results in the different skyrmion numbers, which is clearly found in Fig. 2j,k.

In the high-field region, the 3*Q*-II state and $n_{\mathrm{sk}}=1$ T-SkX are replaced by the other topologically trivial triple-*Q* states depending on $\Gamma$: the triple-*Q* III (3*Q*-III) or the triple-*Q* chiral (3*Q*-Ch) state. The 3*Q*-III state is mainly characterized by the in-plane double-*Q* peaks in Fig. 2f, while the 3*Q*-Ch state is by the in-plane triple-*Q* peaks with equal intensities in Fig. 2g. The 3*Q*-Ch state exhibits nonzero $\chi _{\mathrm{sc}}$, although the skyrmion number becomes zero.

We show *H* dependences of *M* and $|\chi _{\mathrm{sc}}|$ for $\Gamma =0.075$ and 0.2 in Fig. 2n. While increasing *H*, jumps of *M* and $\chi _{\mathrm{sc}}$ appear when the skyrmion number changes: Two jumps between the $n_{\mathrm{sk}}=1$ T-SkX and the 3*Q*-I are found at $H\simeq 0.7$ and $\simeq 0.9$ for $\Gamma =0.075$ and other two jumps are found between the $n_{\mathrm{sk}}=2$ SkX and the $n_{\mathrm{sk}}=1$ T-SkX and between the $n_{\mathrm{sk}}=1$ T-SkX and the 3*Q*-Ch for $\Gamma =0.2$. The transition between the $n_{\mathrm{sk}}=1$ T-SkX and the 3*Q*-III for $0.1<\Gamma <0.17$ also shows jumps of *M* and $\chi _{\mathrm{sc}}$.

### Mechanism of the topological transition

Next, we show the transformation of the skyrmion numbers on the basis of the phase degrees of freedom among the constituent triple-*Q* density waves. We find that the spin configurations for the $n_{\mathrm{sk}}=1$ T-SkX in Fig. 2e and $n_{\mathrm{sk}}=2$ SkX in Fig. 2d are summarized in a single expression as

7 $$\begin{aligned} {\varvec{S}}_i\propto \left( \begin{array}{ccc} -\frac{\sqrt{3}}{2}\sin \mathcal {Q}_{2i}'+\frac{\sqrt{3}}{2}\sin \mathcal {Q}_{3i}' \\ \sin \mathcal {Q}_{1i}' -\frac{1}{2}\sin \mathcal {Q}_{2i}'-\frac{1}{2}\sin \mathcal {Q}_{3i}'\\ A(\cos \mathcal {Q}_{1i}+\cos \mathcal {Q}_{2i}+\cos \mathcal {Q}_{3i})+m_z \end{array}\right) ^T, \end{aligned}$$

where *A* and $m_z$ are additional variational parameters compared to Eq. (1), and we set ${\varvec{e}}_\eta \parallel {\varvec{e}}_z\times {\varvec{Q}}_\eta$ and $\psi \equiv \psi _1=\psi _2=\psi _3$ for $\Gamma >0$. There are two types of phase degrees of freedom in Eq. (7). One is a phase among the constituent waves, $\phi _\eta$, which induces the transformation between the SkX and the vortex crystal with staggered spin scalar chirality^45^. The other is a relative phase between in-plane- and *z*-spin components, $\psi$, which induces the different types of the SkX, as discussed in the introduction: the $n_{\mathrm{sk}}=1$ T-SkX for $0<\psi < \psi _{\mathrm{c}}$ and $n_{\mathrm{sk}}=2$ SkX for $\psi _{\mathrm{c}}\le \psi \le \pi /2$, where $\psi _{\mathrm{c}}$ depends on the other variational parameters. From the symmetry viewpoint, nonzero $\psi$ in the $n_{\mathrm{sk}}=2$ SkX and $n_{\mathrm{sk}}=1$ T-SkX shows the sixfold-rotational symmetry breaking in addition to the inversion symmetry breaking, which is in contrast to $\psi =0$ in the $n_{\mathrm{sk}}=1$ SkX in chiral magnets.

**Figure 3:**
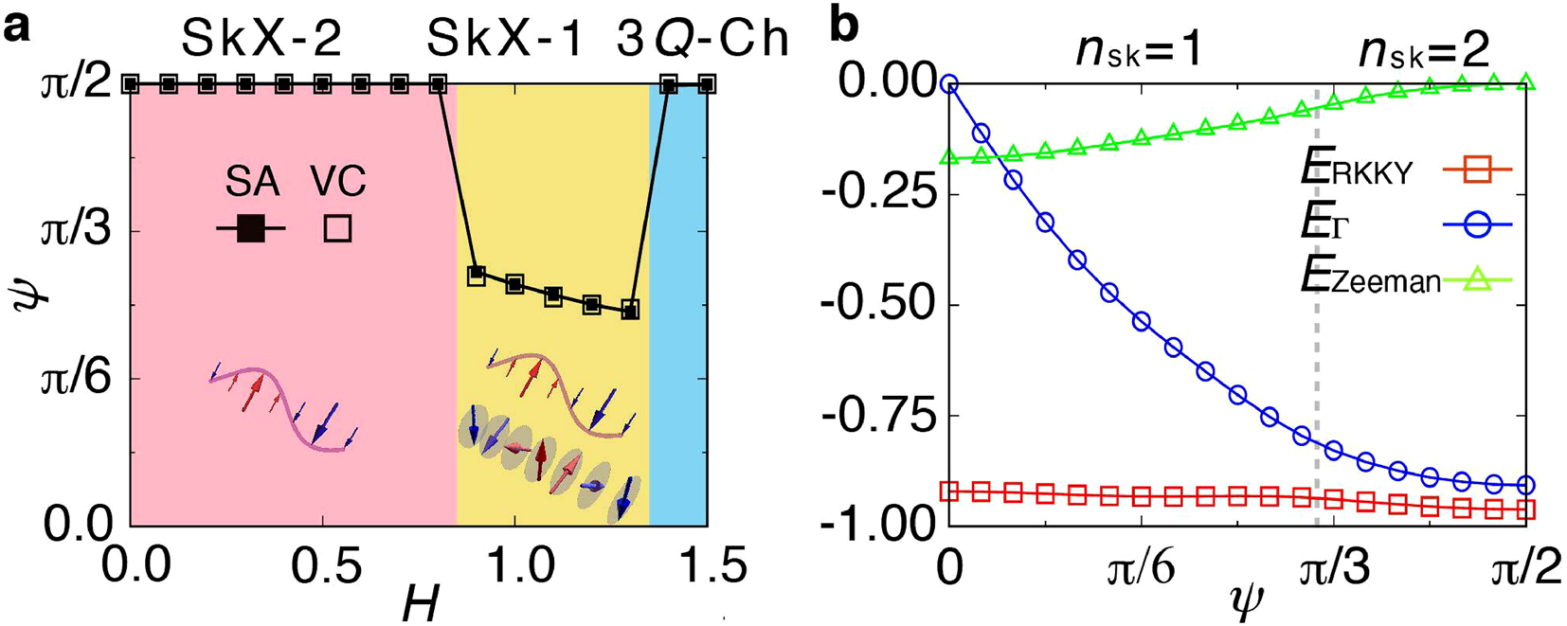
Mechanism of the topological transition for $\Gamma \gtrsim 0.2$. (**a**) *H* dependence of the phase $\psi$ obtained from the simulated annealing (SA) and variational calculation (VC) in the model in Eq. (6) at $\Gamma =0.2$. Schematic pictures of the constitute waves in each SkX are shown in the inset. (**b**) $\psi$ dependences of energies from different contributions, $E_{\mathrm{RKKY}}$, $E_{\Gamma }$, and $E_{\mathrm{Zeeman}}$ in the unit of *J*, $\Gamma$, and *H*, respectively, for $A=1/\sqrt{2}$, $m_z=0$, $\phi _1=4\pi /3$, $\phi _2=2\pi /3$, and $\phi _3=\pi$ in Eq. (7) with $|{\varvec{S}}_i|=1$. The vertical dashed line represents the boundary between the SkXs with $n_{\mathrm{sk}}=1$ and 2.

The phase diagram obtained by the simulated annealing in Fig. 2a is well reproduced by the variational spin ansatz in Eq. (7) for $\Gamma \gtrsim 0.2$. This means that $\psi$ is evaluated through the spin ansatz in Eq. (7). Figure 3a shows *H* dependence of $\psi$ by simulated annealing and variational calculation at $\Gamma =0.2$. Both the results show that the $n_{\mathrm{sk}}=2$ SkX exhibits $\psi =0.5 \pi$, the $n_{\mathrm{sk}}=1$ T-SkX exhibits $0.24\pi \lesssim \psi \lesssim 0.3\pi$, and the 3*Q*-Ch exhibits $\psi =0.5\pi$. Thus, the phase transitions among the $n_{\mathrm{sk}}=2$ SkX, the $n_{\mathrm{sk}}=1$ T-SkX, and the 3*Q*-Ch are regarded as the phase transitions with respect to $\psi$. In other words, the topological transitions are caused by the changes of the types of constitute waves, as shown in Fig. 3a.

The phase transition characterized by the change of $\psi$ is due to the mirror symmetry breaking in the lattice structure. To demonstrate that, we calculate the energy contributions from each term in the model in Eq. (6): the RKKY energy $E_{\mathrm{RKKY}}=-2J\sum _{\eta }{\varvec{S}}_{ {\varvec{Q}}_\eta } \cdot {\varvec{S}}_{- {\varvec{Q}}_\eta }/N$, the anisotropic energy $E_{\Gamma }=-2\Gamma \sum _{\eta \alpha \beta } I^{\alpha \beta }_{{{\varvec{Q}}_\eta }}S^{\alpha }_{{\varvec{Q}}_{\eta }}S^{\beta }_{-{\varvec{Q}}_{\eta }}/N$, and the Zeeman energy $E_{\mathrm{Zeeman}}=-H\sum _i S_i^z/N$. Figure 3b shows $\psi$ dependences of $E_{\mathrm{RKKY}}$, $E_{\Gamma }$, and $E_{\mathrm{Zeeman}}$ for the spin ansatz in Eq. (7) with $|{\varvec{S}}_i|=1$ at fixed $A=1/\sqrt{2}$, $m_z=0$, $\phi _1=4\pi /3$, $\phi _2=2\pi /3$, and $\phi _3=\pi$, where $\psi _c$ is around $\pi /3$. $E_{\mathrm{RKKY}}$ has little $\psi$ dependence, whereas $E_{\Gamma }$ and $E_{\mathrm{Zeeman}}$ show distinct behaviors against $\psi$; $E_{\Gamma }$ ($E_{\mathrm{Zeeman}}$) decreases while increasing (decreasing) $\psi$. In other words, $\Gamma$ arising from the mirror symmetry breaking tends to favor the $n_{\mathrm{sk}}=2$ SkX with $\psi =\pi /2$, while the magnetic field tends to favor the $n_{\mathrm{sk}}=1$ SkX with $\psi =0$. The competition between these distinct behaviors causes the filed-induced transition from the $n_{\mathrm{sk}}=2$ SkX to the $n_{\mathrm{sk}}=1$ T-SkX with $0.24\pi \lesssim \psi \lesssim 0.3\pi$.

## Summary

In conclusion, we clarify that the magnetic anisotropy arising from the breaking of the mirror symmetry is another way to stabilize the SkXs in itinerant magnets irrespective of the spatial inversion symmetry. On the basis of simulated annealing and variational calculation, we show that the anisotropic RKKY interaction induces two SkXs with different topological numbers, which accompanies the spontaneous inversion symmetry breaking. Moreover, we find that two SkXs are transformed with each other by changing the anisotropic RKKY interaction and magnetic field, the former of which is tuned by the degree of mirror symmetry breaking.

Our study reveals that the $n_{\mathrm{sk}}=1$ and $n_{\mathrm{sk}}=2$ SkXs are stabilized even without the multi-spin interaction in itinerant magnets, which is distinct from the previous one in the Kondo lattice model without the magnetic anisotropy^23^: The anisotropic bilinear exchange interaction plays an important role in the stabilization of the former SkXs, while the isotropic biquadratic interaction is important for the latter one^24^. Although both the systems exhibit similar skyrmion textures, the degeneracy in terms of the vorticity and helicity is different owing to the different mechanisms. The SkXs by the isotropic biquadratic interaction are energetically degenerate for different vorticity and helicity, while the present SkXs have a definite vorticity and helicity depending on the sign of the anisotropic interaction. Reflecting such a difference, the SkXs in the present model induce a different Goldstone mode from that in the previous model, which results in different dynamics. Furthermore, the anisotropic response against the electromagnetic field is anticipated due to the nature of magnetic anisotropy, which might give rise to further unconventional multiple-*Q* states. Such a theoretical exploration of the SkXs based on magnetic anisotropy will be left for future study.

Finally, let us discuss a relevant material in the present mechanism. The centrosymmetric itinerant magnet Gd$_2$PdSi$_3$^36^ might be a candidate material, which hosts the skyrmion crystal in an external magnetic field. The importance of the RKKY interaction from the nesting of the Fermi surfaces has already been suggested by the angle-resolved photoemission spectroscopy^46,47^. In addition, the anisotropic RKKY interaction would play an important role in this compound, as the magnetic Gd ions form the triangular lattice and they are sandwiched by the nonmagnetic Pd and Si so that the mirror symmetry on each magnetic layer is broken^39,48^. Indeed, the importance of the multi-orbital degrees of freedom, which become the microscopic origin of the anisotropic RKKY interaction, has also been implied by first-principle calculations^46,49^. It would be interesting to test our scenario for the SkX in Gd$_2$PdSi$_3$ by considering the superstructure of the Pd and Si and the effect of the spin-orbit coupling. Our mechanism will also shed light on engineering the SkXs in quasi-two-dimensional magnetic materials including surface, domain, and layered systems.

## Methods

### Simulated annealing

We perform the simulated annealing combined with the standard Metropolis local updates under the periodic boundary condition. In the simulation, we gradually reduce the temperature with a rate $T_{n+1}=\alpha T_n$, where $T_n$ is the temperature at the *n*th step. We set the initial temperature $T_0=1$ and the coefficient $\alpha =0.99954$. The final temperature $T\simeq 0.01$ is reached after total $10^6$ steps, where we perform $10^2$ Monte Carlo sweeps at each temperature. At the final temperature, we perform $10^6$ Monte Carlo sweeps for thermalization and measurements, respectively. Although Figs. 2 and 3a show the results for $N=48^2$, we confirm that the obtained result does not change for $N=96^2$. We also confirm that the simulations with different values of $\alpha$, $\alpha =0.99908, 0.99541$, and 0.95499, give the same result.

### Variational calculation

We here present the details of the spin ansatz in Eq. (7) and the variational calculation. To find the spin configuration in Eq. (7), we start from a general spin ansatz of the single-*Q* spiral state given by

8 $$\begin{aligned} {\varvec{S}}_i^{\eta }&=\tilde{{\varvec{e}}}_{\eta y}b\sin \mathcal {Q}_{\eta i}''+\tilde{{\varvec{e}}}_{\eta z}a\cos \mathcal {Q}_{\eta i}'', \end{aligned}$$

where $\mathcal {Q}_{\eta i}''={\varvec{Q}}_\eta \cdot {\varvec{r}}_i+\varphi _\eta$, $\tilde{{\varvec{e}}}_{\eta y}={\varvec{e}}_\eta \cos \theta -{\varvec{e}}_z\sin \theta$, and $\tilde{{\varvec{e}}}_{\eta z}={\varvec{e}}_\eta \sin \theta +{\varvec{e}}_z\cos \theta$ ($0\le \theta <\pi$). This spin configuration expresses an elliptical wave, where the axis with a length of 2*a* is parallel to $\tilde{{\varvec{e}}}_{\eta z}$ and the axis with a length of 2*b* ($a>b\ge 0$) is parallel to $\tilde{{\varvec{e}}}_{\eta y}$. The spin ansatz in Eq. (8) describes a variety of spin density waves depending on $r\equiv b/a$ and $\theta$: the spiral wave ($r=1$), standard elliptical wave ($0<r<1$ and $\theta =0,\pi /2$), rotated elliptical wave ($0<r<1$ and $\theta \ne 0,\pi /2$), standard sinusoidal wave ($r=0$ and $\theta =0,\pi /2$), and rotated sinusoidal wave ($r=0$ and $\theta \ne 0,\pi /2$), where “standard” means that the axes are parallel to ${\varvec{e}}_\eta$ or ${\varvec{e}}_z$. A schematic picture of spiral plane in Eq. (8) is shown in Supplementary Information.

We rewrite the spin configuration in Eq. (8) as

9 $$\begin{aligned} {\varvec{S}}_i^{\eta }&={\varvec{e}}_{\eta }A_{\perp }\sin (\mathcal {Q}_{\eta i}''+\Theta _\perp )+{\varvec{e}}_{z} A_z\cos (\mathcal {Q}_{\eta i}''+\Theta _z), \end{aligned}$$

where $\Theta _\perp$, $\Theta _z$, $A_z,$ and $A_\perp$ satisfy $\tan \Theta _\perp =\tan \theta /r$, $\tan \Theta _z=r\tan \theta$, $A_\perp =\sqrt{a^2\sin ^2\theta +b^2\cos ^2\theta }$, and $A_z=\sqrt{a^2\cos ^2\theta +b^2\sin ^2\theta }$, respectively. Then, the spin ansatz in Eq. (7) is obtained by superposing three spin density waves in Eq. (9) in addition to a uniform *z* component, which is given by

10 $$\begin{aligned} {\varvec{S}}_i\propto \sum _\eta {\varvec{S}}_i^{\eta }+M_z{\varvec{e}}_z. \end{aligned}$$

The variational parameters *A*, $m_z$, $\phi _\eta$, and $\psi _\eta$ in Eq. (7) are related to that in Eq. (10) as $A=A_z/A_\perp$, $m_z=M_z/A_\perp$ $\phi _\eta =\varphi _\eta +\Theta _z$ and $\psi _\eta =\Theta _\perp -\Theta _z$. From the definition, $\psi _\eta$ does not depend on $\eta$, i.e., $\psi _1=\psi _2=\psi _3 \equiv \psi$.

In the variational calculations in Fig. 3a, we optimize *a*, *b*, $\theta$, $\varphi _\eta$, and $M_z$ as the variational parameters for $N=12^2$. After obtaining the optimal parameters, we calculate $\psi$ in Fig. 3a from the difference between phases of $S^{y}_{{\varvec{Q}}_1}$ and $S^{z}_{{\varvec{Q}}_1}$. In Supplementary Information, we show *H* dependences of the magnetic moment, magnetization, and spin scalar chirality by the variational calculation and the simulated annealing. Compared to the results, one can find that the variational spin ansatz in Eq. (7) corresponds to the spin textures obtained by the simulated annealing.

## Supplementary Information

Supplementary Information.

## Data Availability

The datasets generated during and/or analysed during the current study are available from the corresponding author on reasonable request.
