# Supplementary material for: Skyrmion crystals in centrosymmetric itinerant magnets without horizontal mirror plane
Source: Sci Rep. 2021 May 27;11:11184. doi: 10.1038/s41598-021-90308-1 (PMC8160153; doi:10.1038/s41598-021-90308-1)
Supplement: Supplementary file 1 — Supplementary Information. [file 41598_2021_90308_MOESM1_ESM.pdf]

Supplementary Information: Skyrmion crystals in centrosymmetric itinerant magnets without horizontal mirror plane

Ryota Yambe[1,2,*] **and Satoru Hayami**[2]

[1]Department of Physics, Hokkaido University, Sapporo 060-0810, Japan
[2]Department of Applied Physics, The University of Tokyo, Tokyo 113-8656, Japan
[*]corresponding.yambe@jphys.t.u-tokyo.ac.jp

Derivation of the effective spin model

We derive the effective spin model in Eq. (2) for the itinerant magnets in the main text. We start from the multi-band periodic Anderson model for the trigonal lattice structure shown in Fig. 1d, which is given by

$$\mathscr{H}_{\rm PA} = \mathscr{H}_0 + \mathscr{H}_V, \tag{S1}$$

$$\mathscr{H}_0 = \sum_{m\boldsymbol{k}\sigma} (\varepsilon_{m\boldsymbol{k}} - \mu) c^{\dagger}_{m\boldsymbol{k}\sigma} c_{m\boldsymbol{k}\sigma} + (E_f - \mu) \sum_{i\sigma} n_{i\sigma} + U \sum_{i} n_{i\uparrow} n_{i\downarrow}, \tag{S2}$$

$$\mathscr{H}_V = \frac{1}{\sqrt{N}} \sum_{mi\boldsymbol{k}\sigma\sigma'} \left\{ e^{i\boldsymbol{k}\cdot\boldsymbol{R}_i} f^{\dagger}_{i\sigma} \left(V^0_{m\boldsymbol{k}} \delta_{\sigma\sigma'} + \boldsymbol{V}_{m\boldsymbol{k}} \cdot \boldsymbol{\sigma}_{\sigma\sigma'} \right) c_{m\boldsymbol{k}\sigma'} + \text{h.c.} \right\}. \tag{S3}$$

Here, $c^{\dagger}_{m\boldsymbol{k}\sigma}$ ($c_{m\boldsymbol{k}\sigma}$) is a creation (annihilation) operator of an itinerant electron with wave vector $\boldsymbol{k}$ and spin σ on a nonmagnetic layer m, $f^{\dagger}_{i\sigma}$ ($f_{i\sigma}$) is the creation (annihilation) operator of a localized f electron at site $\boldsymbol{R}_i$ with spin σ on a magnetic layer, $n_{i\sigma} = f^{\dagger}_{i\sigma} f_{i\sigma}$, N is the number of lattice sites, and h.c. represents the Hermitian conjugate. The total Hamiltonian $\mathscr{H}_{\rm PA}$ consists of the non-hybridized Hamiltonian $\mathscr{H}_0$ and the hybridized Hamiltonian $\mathscr{H}_V$. For $\mathscr{H}_0$, $\varepsilon_{m\boldsymbol{k}}$ and E_f are the energy dispersion for the itinerant electrons and the atomic energy for the localized f electrons, respectively. μ is the chemical potential. U is the Hubbard-type Coulomb repulsion between f electrons with opposite spins on the same site. For $\mathscr{H}_V$, $V_{m\boldsymbol{k}}\delta_{\sigma\sigma'}$ is the spin-independent c-f hybridization, where $\delta_{\sigma\sigma'}$ is the Kronecker delta, while $\boldsymbol{V}_{m\boldsymbol{k}} \cdot \boldsymbol{\sigma}_{\sigma\sigma'} = \sum_{\alpha=x,y,z} V^{\alpha}_{m\boldsymbol{k}} \sigma^{\alpha}_{\sigma\sigma'}$ is the spin-dependent c-f hybridization, where $\boldsymbol{\sigma}_{\sigma\sigma'} = (\sigma^x, \sigma^y, \sigma^z)_{\sigma\sigma'}$ are the Pauli matrices. The spin-dependent c-f hybridization arises from the mixture of up- and down-spin basis functions owing to the strong spin-orbit coupling in the localized f electron.

We here consider the situation where the localized f-orbital levels located at E_f and $E_f + U$ are far away from the Fermi level. In this situation, the f electron is approximately treated as the localized spin. For the small but nonzero c-f hybridizations, the two degenerate localized states are mixed via electron hopping, where the hybridized Hamiltonian $\mathscr{H}_V$ is the perturbation term. Then, a low-energy effective model is obtained by using the Schrieffer-Wolff transformation[1] represented by

$$\begin{aligned} \bar{\mathscr{H}} &= e^{\mathscr{S}} \mathscr{H}_{\rm PA} e^{-\mathscr{S}} \\ &= \mathscr{H}_0 + \mathscr{H}_V + [\mathscr{S}, \mathscr{H}_0] + [\mathscr{S}, \mathscr{H}_V] + \frac{1}{2} [\mathscr{S}, [\mathscr{S}, \mathscr{H}_0]] + \cdots, \end{aligned} \tag{S4}$$

where $[A,B] = AB - BA$. We choose the generator $\mathscr{S}$ satisfying $\mathscr{H}_V + [\mathscr{S}, \mathscr{H}_0] = 0$ as

$$\mathscr{S} = \frac{1}{\sqrt{N}} \sum_{mi\boldsymbol{k}\sigma\sigma'} (A_{m\boldsymbol{k}} + B_{m\boldsymbol{k}} n_{i\bar{\sigma}}) \left\{ e^{i\boldsymbol{k}\cdot\boldsymbol{R}_i} f^{\dagger}_{i\sigma} \left(V^0_{m\boldsymbol{k}} \delta_{\sigma\sigma'} + \boldsymbol{V}_{m\boldsymbol{k}} \cdot \boldsymbol{\sigma}_{\sigma\sigma'} \right) c_{m\boldsymbol{k}\sigma'} - \text{h.c.} \right\}, \tag{S5}$$

$$A_{m\boldsymbol{k}} = \frac{1}{E_f - \varepsilon_{m\boldsymbol{k}}}, \tag{S6}$$

$$B_{m\boldsymbol{k}} = \frac{1}{\varepsilon_{m\boldsymbol{k}} - E_f} - \frac{1}{\varepsilon_{m\boldsymbol{k}} - E_f - U}, \tag{S7}$$

so that the first-order contribution from $\mathscr{H}_V$ becomes zero. Then, $\bar{\mathscr{H}}$ can be approximately expressed as

$$\bar{\mathscr{H}} = \mathscr{H}_0 + \frac{1}{2} [\mathscr{S}, \mathscr{H}_V]. \tag{S8}$$

One can obtain the Kondo lattice model from Eq. (S8) under the condition that $\sum_\sigma n_{i\sigma} = 1$ as

$$H_{\rm KL} = H_0 + H', \tag{S9}$$

$$H_0 = \sum_{m\boldsymbol{k}\sigma} (\varepsilon_{m\boldsymbol{k}} - \mu) c^\dagger_{m\boldsymbol{k}\sigma} c_{m\boldsymbol{k}\sigma}, \tag{S10}$$

$$H' = \frac{1}{\sqrt{N}} \sum_{m\boldsymbol{k}\boldsymbol{q}\sigma\sigma'} \sum_{\alpha\beta} J^{\alpha\beta}_{m\boldsymbol{k}+\boldsymbol{q}\boldsymbol{k}} c^\dagger_{m\boldsymbol{k}+\boldsymbol{q}\sigma} \sigma^\alpha_{\sigma\sigma'} c_{m\boldsymbol{k}\sigma'} S^\beta_{\boldsymbol{q}}, \tag{S11}$$

where $J^{\alpha\beta}_{m\boldsymbol{k}'\boldsymbol{k}}$ $(\alpha, \beta = x, y, z)$ is a generalized spin-charge coupling[2]. Note that we neglect contributions from the spin-dependent energy dispersion for itinerant electrons in Eq. (S10), the spin-independent and spin-dependent hybridizations between itinerant electrons on different nonmagnetic layers in Eq. (S10), and the hybridizations between itinerant electrons on different nonmagnetic layers via $\boldsymbol{S}_{\boldsymbol{q}}$ in Eq. (S11). The obtained spin-charge coupling $J^{\alpha\beta}_{m\boldsymbol{k}'\boldsymbol{k}}$ is decomposed into isotropic ($J^{\rm ISO}_{m\boldsymbol{k}'\boldsymbol{k}}$), symmetric anisotropic ($J^{\rm SA}_{m\boldsymbol{k}'\boldsymbol{k}}$), and antisymmetric Dzyaloshinskii-Moriya-type ($J^{\rm DM}_{m\boldsymbol{k}'\boldsymbol{k}}$) spin-charge couplings as

$$J^{\alpha\beta}_{m\boldsymbol{k}'\boldsymbol{k}} = J^{\rm ISO}_{m\boldsymbol{k}'\boldsymbol{k}} \delta_{\alpha\beta} + \left[J^{\rm SA}_{m\boldsymbol{k}'\boldsymbol{k}}\right]^{\alpha\beta} + \left[J^{\rm DM}_{m\boldsymbol{k}'\boldsymbol{k}}\right]^{\alpha\beta}, \tag{S12}$$

where

$$J^{\rm ISO}_{m\boldsymbol{k}'\boldsymbol{k}} = \frac{B_{m\boldsymbol{k}} + B_{m\boldsymbol{k}'}}{2} \left(V^0_{m\boldsymbol{k}} V^{0*}_{m\boldsymbol{k}'} - V^x_{m\boldsymbol{k}} V^{x*}_{m\boldsymbol{k}'} - V^y_{m\boldsymbol{k}} V^{y*}_{m\boldsymbol{k}'} - V^z_{m\boldsymbol{k}} V^{z*}_{m\boldsymbol{k}'} \right), \tag{S13}$$

$$[J^{\rm SA}_{m\boldsymbol{k}'\boldsymbol{k}}]^{\alpha\beta} = \frac{B_{m\boldsymbol{k}} + B_{m\boldsymbol{k}'}}{2} \left(V^\alpha_{m\boldsymbol{k}} V^{\beta*}_{m\boldsymbol{k}'} + V^{\alpha*}_{m\boldsymbol{k}'} V^\beta_{m\boldsymbol{k}} \right), \tag{S14}$$

$$[J^{\rm DM}_{m\boldsymbol{k}'\boldsymbol{k}}]^{\alpha\beta} = \frac{B_{m\boldsymbol{k}} + B_{m\boldsymbol{k}'}}{2} i \sum_{\gamma=x,y,z} \varepsilon_{\alpha\beta\gamma} (V^\gamma_{m\boldsymbol{k}} V^{0*}_{m\boldsymbol{k}'} - V^{\gamma*}_{m\boldsymbol{k}'} V^0_{m\boldsymbol{k}}). \tag{S15}$$

Here, $\varepsilon_{\alpha\beta\gamma}$ is the Levi-Civita symbol.

We derive an effective spin model for the Kondo lattice model in Eq. (S9) by expanding the grand potential Ω with respect to the generalized spin-charge coupling $J^{\alpha\beta}_{m\boldsymbol{k}'\boldsymbol{k}}$ [3,4]. The grand potential is expanded as

$$\begin{aligned}
\Omega - \Omega^{(0)} &= -\frac{1}{\beta} \ln \left\langle \mathscr{T}_\tau \exp\left(-\int_0^\beta d\tau H'_\tau \right) \right\rangle_0 \\
&= -\frac{1}{\beta} \sum_{n=1}^\infty \frac{1}{(2n)!} \int_0^\beta d\tau_1 \cdots \int_0^\beta d\tau_{2n} \langle \mathscr{T}_\tau H'_{\tau_1} \cdots H'_{\tau_{2n}} \rangle_{\rm con} \\
&= \sum_{n=1}^\infty \Omega^{(2n)},
\end{aligned} \tag{S16}$$

where $\Omega^{(0)}$ represents the grand potential from H_0, β is the inverse temperature, $\langle \cdots \rangle_0 \equiv {\rm Tr}(e^{-\beta H_0} \cdots)/{\rm Tr} e^{-\beta H_0}$, $\mathscr{T}_\tau$ is the time ordered product, τ is the imaginary time, $H'_\tau = e^{\tau H_0} H' e^{-\tau H_0}$, and $\langle \cdots \rangle_{\rm con}$ represents contributions from the connected Feynman diagrams. Note that odd contributions vanish due to the time-reversal symmetry.

The second-order contribution is given by

$$\begin{aligned}
\Omega^{(2)} &= -\frac{1}{2!\beta} \int_0^\beta d\tau_1 \int_0^\beta d\tau_2 \langle \mathscr{T}_\tau H'_{\tau_1} H'_{\tau_2} \rangle_{\rm con} \\
&= \frac{1}{\beta N} \sum_{m\boldsymbol{k}\boldsymbol{q}} \sum_{\alpha\beta\gamma} \sum_{\omega_n} \mathscr{G}_{m\boldsymbol{k}+\boldsymbol{q}}(i\omega_n) \mathscr{G}_{m\boldsymbol{k}}(i\omega_n) J^{\gamma\alpha}_{m\boldsymbol{k}+\boldsymbol{q}\boldsymbol{k}} J^{\gamma\beta}_{m\boldsymbol{k}\boldsymbol{k}+\boldsymbol{q}} S^\alpha_{\boldsymbol{q}} S^\beta_{-\boldsymbol{q}},
\end{aligned} \tag{S17}$$

where $\mathscr{G}_{m\boldsymbol{k}}(i\omega_n) = 1/(i\omega_n - \varepsilon_{m\boldsymbol{k}} + \mu)$ is the noninteracting Green's function with the Matsubara frequency ω_n. It is noted that the Green's function does not depend on the spin and its spin dependence is omitted for notational simplicity. By taking the summation of ω_n, the second-order contribution is given by

$$\Omega^{(2)} = -\sum_{\boldsymbol{q}} \sum_{\alpha\beta} \chi^{\alpha\beta}_{\boldsymbol{q}} S^\alpha_{\boldsymbol{q}} S^\beta_{-\boldsymbol{q}}, \tag{S18}$$

$$\chi^{\alpha\beta}_{\boldsymbol{q}} = \frac{1}{N} \sum_{m\boldsymbol{k}} \sum_{\gamma} \frac{f(\varepsilon_{m\boldsymbol{k}}) - f(\varepsilon_{m\boldsymbol{k}+\boldsymbol{q}})}{\varepsilon_{m\boldsymbol{k}+\boldsymbol{q}} - \varepsilon_{m\boldsymbol{k}}} J^{\gamma\alpha}_{m\boldsymbol{k}+\boldsymbol{q}\boldsymbol{k}} J^{\gamma\beta}_{m\boldsymbol{k}\boldsymbol{k}+\boldsymbol{q}}. \tag{S19}$$

Here, $f(\varepsilon_{m\boldsymbol{k}})$ is the Fermi distribution function, and $\chi_{\boldsymbol{q}}^{\alpha\beta}$ divided by the square of the energy scale of the spin-charge coupling corresponds to the bare susceptibility of itinerant electrons.

The effective spin model in Eq. (6) is obtained by considering an appropriate orbital in Eq. (S1). For example, when we consider the f orbital in the functional form of $z(5z^2-3r^2)$ on a magnetic layer and the s orbital on nonmagnetic layers, we obtain the nonzero Γ contributions via nonzero $V^0_{m\boldsymbol{k}}$, $V^x_{m\boldsymbol{k}}$, and $V^y_{m\boldsymbol{k}}$: $K^{yz}_{\boldsymbol{Q}_1} = K^{zy}_{\boldsymbol{Q}_1} = -2K^{xz}_{\boldsymbol{Q}_2}/\sqrt{3} = -2K^{zx}_{\boldsymbol{Q}_2}/\sqrt{3} = -2K^{yz}_{\boldsymbol{Q}_2} = -2K^{zy}_{\boldsymbol{Q}_2} = 2K^{xz}_{\boldsymbol{Q}_3}/\sqrt{3} = 2K^{zx}_{\boldsymbol{Q}_3}/\sqrt{3} = -2K^{yz}_{\boldsymbol{Q}_3} = -2K^{zy}_{\boldsymbol{Q}_3}$. It is noted that there are other nonzero anisotropic components for $\boldsymbol{Q}_\eta$ to satisfy the D_{3d} point group symmetries, e.g., diagonal components $K^{\alpha\alpha}_{\boldsymbol{Q}_\eta}$ and off-diagonal components $K^{xy}_{\boldsymbol{Q}_{\eta=2,3}}$ [5,6]. In the main text, however, we omit such components in the present model to focus on the effect on the multiple-Q nature induced by the mirror symmetry breaking.

Variational calculation

We here present the details of the variational calculation. Figure S1a shows a schematic picture of spiral plane in Eq. (8), which clearly shows a variety of spin density waves described in Eq. (8): the spiral wave ($r \equiv b/a = 1$), standard elliptical wave ($0 < r < 1$ and $\theta = 0, \pi/2$), rotated elliptical wave ($0 < r < 1$ and $\theta \neq 0, \pi/2$), standard sinusoidal wave ($r = 0$ and $\theta = 0, \pi/2$), and rotated sinusoidal wave ($r = 0$ and $\theta \neq 0, \pi/2$), where "standard" means that the axes are parallel to $\boldsymbol{e}_\eta$ or $\boldsymbol{e}_z$.

Figures S1b and S1c show H dependences of the magnetic moment $m^\alpha_{\boldsymbol{q}} = \sqrt{S^{\alpha\alpha}_s(\boldsymbol{q})/N}$, magnetization M, and spin scalar chirality $\chi_{\rm sc}$ by the variational calculation for $N = 12^2$ and the simulated annealing. Compared to the results, one can find that the variational spin ansatz in Eq. (7) in the main text corresponds to the spin textures obtained by the simulated annealing.

Figure S1d shows the optimal r and θ in Eq. (8) denoted as $r_{\rm opt}$ and $\theta_{\rm opt}$. The result clearly shows the types of spin density waves: the rotated sinusoidal wave ($r_{\rm opt} = 0$) in SkX-2 and 3Q-Ch, and the rotated elliptical wave ($0.22 < r_{\rm opt} < 0.26$) in SkX-1, where the axis with a length of $2a$ is rotated from the $\boldsymbol{e}_z$ axis ($0.25\pi < \theta_{\rm opt} < 0.38\pi$). A difference between the SkX-2 and 3Q-Ch state is found in A and m_z in Eq. (7). The SkX-2 is characterized by large A/m_z and the 3Q-Ch state is by small A/m_z. As a result, the z-spin component in the 3Q-Ch state is positive, which indicates no skyrmion number. It is noted that the rotated axis is important to induce the SkX-1, SkX-2 and 3Q-Ch, as the spin configuration in Eq. (7) with standard elliptical (sinusoidal) waves expresses the $n_{\rm sk} = 1$ SkX with $\psi = 0$ [the collinear ($\theta = 0$) or the coplanar ($\theta = \pi/2$) state].

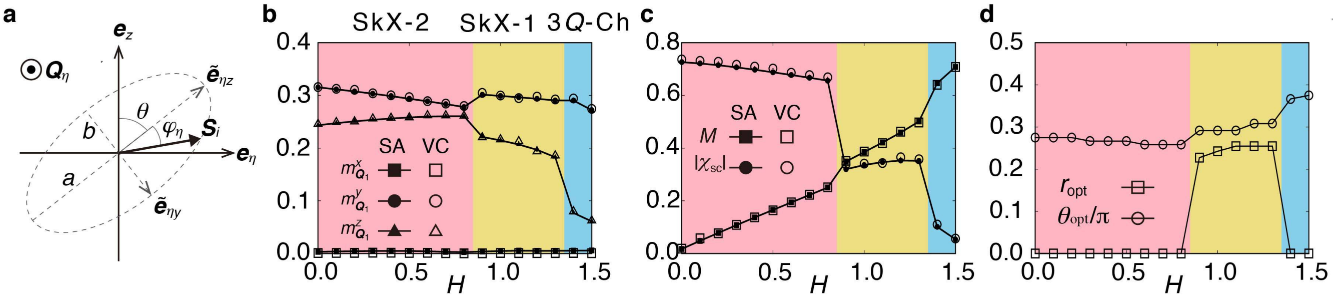

Figure S1. Details of the variational calculation. **a** Schematic picture of spiral plane for the spin density wave in Eq. (8). **b** H dependences of the magnetic moment $m^\alpha_{\boldsymbol{Q}_1}$ and **c** magnetization M and spin scalar chirality $|\chi_{\rm sc}|$ obtained by simulated annealing (SA) and variational calculation (VC) at $\Gamma = 0.2$. **d** H dependences of the ratio of the length of the axes $r_{\rm opt}$ and the angle $\theta_{\rm opt}$ obtained by the variational calculation.

References

1. Schrieffer, J. R. & Wolff, P. A. Relation between the Anderson and Kondo Hamiltonians. *Phys. Rev.* **149**, 491–492, DOI: https://doi.org/10.1103/PhysRev.149.491 (1966).

2. Xia, K., Zhang, W., Lu, M. & Zhai, H. Noncollinear interlayer exchange coupling caused by interface spin-orbit interaction. *Phys. Rev. B* **55**, 12561–12565, DOI: https://doi.org/10.1103/PhysRevB.55.12561 (1997).

3. Akagi, Y., Udagawa, M. & Motome, Y. Hidden Multiple-Spin Interactions as an Origin of Spin Scalar Chiral Order in Frustrated Kondo Lattice Models. *Phys. Rev. Lett.* **108**, 096401, DOI: https://doi.org/10.1103/PhysRevLett.108.096401 (2012).

4. Hayami, S., Ozawa, R. & Motome, Y. Effective bilinear-biquadratic model for noncoplanar ordering in itinerant magnets. *Phys. Rev. B* **95**, 224424, DOI: https://doi.org/10.1103/PhysRevB.95.224424 (2017).

5. Hayami, S. & Motome, Y. Noncoplanar multiple-q spin textures by itinerant frustration: Effects of single-ion anisotropy and bond-dependent anisotropy. *Phys. Rev. B* **103**, 054422, DOI: https://doi.org/10.1103/PhysRevB.103.054422 (2021).

6. Hayami, S. Multiple-Q magnetism by anisotropic bilinear-biquadratic interactions in momentum space. *J. Magn. Magn. Mater.* **513**, 167181, DOI: https://doi.org/10.1016/j.jmmm.2020.167181 (2020).
